# Exploration of Rapid Adaptation of First Contact Physiotherapy Services During the COVID‐19 Pandemic: A Three‐Phase Sequential Mixed‐Methods Study Protocol

**DOI:** 10.1002/hsr2.70653

**Published:** 2025-04-18

**Authors:** Oluwatoyin Adenike Adeniji, Nicola Carey, Evangelos Pappas, Victoria Traynor, Karen Stenner, Theopisti Chrysanthaki

**Affiliations:** ^1^ School of Health Sciences, Faculty of Health and Medical Sciences University of Surrey Guildford England UK; ^2^ School of Medical, Indigenous and Health Sciences, Faculty of Science Medicine and Health University of Wollongong Wollongong New South Wales Australia; ^3^ Centre for Rural Health Sciences University of the Highlands and Islands Inverness Scotland UK; ^4^ School of Health and Biomedical Sciences Royal Melbourne Institute of Technology University Melbourne Victoria Australia; ^5^ Sydney School of Health Sciences The University of Sydney Camperdown New South Wales Australia; ^6^ School of Health University of Sunshine Coast Sippy Downs Queensland Australia

**Keywords:** COVID‐19 pandemic, First contact physiotherapy, Healthcare service delivery, Musculoskeletal, Rapid adaptation, Rapid implementation

## Abstract

**Background:**

The COVID‐19 pandemic significantly disrupted primary care and emergency departments globally, including the UK and Australia respectively, affecting services within these settings, such as first contact physiotherapy services (FCPS) for patients with musculoskeletal conditions. This disruption necessitated rapid adaptation to ensure continuity of care. Before this study, comprehensive adaptations across both primary care and emergency departments FCPS had not been documented. Additionally, the specific adaptation processes, strategies used, and experiences of both staff and patients during the COVID‐19 pandemic were unclear. Variations in responses between the UK and Australia also remained unknown. Documenting these rapid adaptations and experiences is crucial for future preparedness, as it provides valuable insights to guide FCPS and similar services, preventing future disruptions and promoting continuity of care. Moreover, findings will contribute significant knowledge to the existing literature.

**Aim:**

This study explores rapid adaptation of FCPS for patients with musculoskeletal conditions during the COVID‐19 pandemic in the UK and Australia.

**Methods:**

This is an ongoing three‐phase sequential mixed‐methods study. Phase 1 utilises a cross‐sectional survey of physiotherapists in FCPS role in the UK and Australia to assess changes in healthcare delivery during the pandemic, as well as levels of readiness and responsiveness, highlighting similarities and differences. Phase 2 employs a case‐study approach, including semi‐structured interviews and a review of documents produced to direct the management and implementation of proposed changes in FCPS, to further understand the findings from Phase 1. In phase 3, mixed‐methods integration facilitates the development of context specific recommendations for the rapid adaptation of FCPS and similar contexts. These recommendations will be presented to experts for feedback and further refinement.

**Discussion:**

The mixed‐methods research will provide contextually rich account of FCPS rapid adaptation, providing key learnings that could be applied to implement evidence‐informed rapid adaptation in FCPS during public health emergencies.

## 1 Introduction

### 1.1 Background

The COVID‐19 pandemic, declared a global health emergency by the World Health Organisation (WHO) in March 2020 [1], significantly impacted healthcare services worldwide, challenging the resilience of healthcare systems [2]. With the highly contagious nature of the COVID‐19 virus, resources were diverted to manage COVID infections, leading to disruptions in essential services [3, 4]. One such affected area was musculoskeletal (MSK) services, provided through first contact physiotherapy services (FCPS), typically at the point of healthcare access in primary care and emergency departments [5, 6]. These services, which play a vital role in assessing and managing MSK conditions experienced significant disruptions [7] necessitating rapid adaptation [8, 9].

Before the COVID‐19 pandemic, the WHO emphasised the importance of healthcare system readiness for public health emergencies to effectively respond to population healthcare needs as a well‐prepared health system was seen as having the ability to adapt and sustain other healthcare services during emergencies [10, 11]. However, the response to the COVID‐19 pandemic exposed gaps in preparedness, with increased demand from COVID‐19 patients leading to disruptions in other healthcare services [2, 3, 4]. To address this, the WHO advocated for rapid adaptation of healthcare services. Rapid adaptation is described as the rapid implementation of changes in healthcare delivery to optimise resource utilisation and address population needs, to mitigate the risk of outright system failure [12]. However, there is paucity of evidence to guide rapid adaptation during public health emergencies, and a lack of agreement about what constitutes ‘rapid’. Also, the absence of clear direction and adequate evidence on rapid adaptation in healthcare service delivery led to trial and error, confusion, and uncertainties globally [4, 13, 14].

In response to this, WHO developed an operational guide for rapid adaptation as part of the WHO COVID‐19 strategic readiness and responsiveness plan to mitigate disruptions in essential healthcare services [15]. Despite efforts to promote rapid adaptation, health services still struggled to adapt their services [4], not unsurprising when faced and unprepared for unprecedented challenges of COVID‐19 pandemic. There is no evidence on the adoption of these guide, including within FCPS where disruptions in service delivery posed significant risks to patients with MSK conditions [16]. This may be due to the need to localise the guide to specific context. As some changes were implemented without robust evidence supporting their effectiveness, WHO suggested that certain adaptations may not be sustainable and may require reversal, while others may be suitable for a temporary period. Those proven effective, safe, and beneficial can be integrated into routine post‐pandemic practice [4]. Consequently, approaches and assessments of service delivery adaptations during public health emergencies, including the COVID‐19 pandemic, have emphasised readiness, responsiveness, and sustainability.

Historically, this approach focused on the acute situations at hand, and COVID‐19 pandemic underscored the necessity of applying similar strategies to essential services in primary care and emergency departments such as FCPS. There is a paucity of studies in this area and no exploration of FCPS using this approach. As disruption in FCPS poses significant risks to patients with MSK conditions, potentially leading to adverse health effects, including reduced quality of life, thereby straining the healthcare system [17, 18], an intervention to mitigate these risks is needed. Understanding the implementation of rapid adaptations in FCPS through the lenses of readiness, responsiveness, and sustainability will provide valuable insights to better prepare FCPS and similar services for future public health emergencies. In addition, it is important to understand the contextual variations for tailored strategies.

To address this, a sequential mixed‐methods study design is being used [19], as it is commonly used in health services research, including physiotherapy [20]. This approach enables quantitative data to build upon qualitative findings, enriching the breadth and depth of understanding of the phenomenon under study. Furthermore, it offers a valuable framework for generating data that informs intervention knowledge, which is crucial for healthcare services, while taking into account the features of the healthcare context in which the intervention was delivered to patients [21].

### 1.2 Study Setting

This study is being conducted in the UK and Australia. They are exemplar countries as both countries have established FCPS with a standardised frameworks for practice and established at varied point of access to healthcare for the public, that is, primary care and emergency departments, respectively [22, 23, 24, 25, 26]. These allows varied contextual learning regarding FCPS rapid adaptation, which may be transferrable to other environments where there are such services. Importantly, both countries had undergone evaluations before the pandemic [6, 27, 28, 29], providing the opportunity to understand service delivery before and during the pandemic.

### 1.3 Aim of the Study

This study explores rapid adaptation of FCPS for patients with MSK conditions during the COVID‐19 pandemic in the UK and Australia.

## 2 Methods

This protocol follows the mixed‐methods reporting in rehabilitation and health sciences guidelines [30].

### 2.1 Study Design

A three‐phase sequential mixed‐methods study design (quantitative to qualitative) is being used to address the research aim and objectives [19] (Table 1). This study is ongoing, with data collection for phases 1 and 2 completed, while phase 3 is in progress.

**Table 1:** Relationship between mixed method phases, research objectives and the data collection methods.

Study phases	S/N	Research objectives	Data collection element
Phase 1 (Quantitative)	1	To identify the time and types of adaptation in FCPS healthcare service delivery in the UK and Australia during COVID‐19 pandemic.	Cross sectional survey
	2	To determine the sustainability of the adaptations in FCPS healthcare service delivery in the UK and Australia during COVID‐19 pandemic.	
	3	To assess the level of readiness and responsiveness of FCPS healthcare service delivery adaptation during COVID‐19 pandemic in the UK and Australia.	
	4	To identify differences in FCPS rapid adaptation in the UK and Australia.	
Phase 2 (Qualitative)	5	To investigate physiotherapists in FCPS role, and other stakeholders' experiences and views, including barriers and facilitators, strategies for adaptations in FCPS healthcare service delivery in the UK and Australia during COVID‐19 pandemic.	Interviews
	6	To review documents from healthcare organisations that detailed the aims, processes, strategies and outcomes of the implemented changes within the FCPS in the UK and Australia during the COVID‐19 pandemic.	Documentary evidence
Phase 3 (Mixed methods integration)	7	To develop a rapid adaptation recommendation to guide FCPS.	Informed by research findings and feedback/recommendations from stakeholders and expert's consultation.

### 2.2 Philosophical Perspective

**Figure 1:**
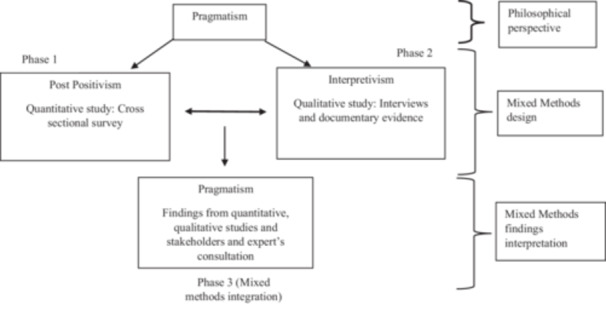
Mixing of methods using pragmatism.

Pragmatism will be used to guide and interpret findings from this study (Figure 1). The pragmatism paradigm aligns well with mixed‐methods designs as it prioritises “what works” by integrating findings from both post‐positivism perspective, which uses objective facts through quantitative studies and interpretivism perspective, which relies on subjective experience obtained from qualitative studies [31].

### 2.3 Sampling and Recruitment

#### 2.3.1 Phase 1: Quantitative (Cross Sectional Survey)

A non‐probability sampling method was employed to recruit physiotherapists practicing in first contact role across the UK and Australia (See Table 2 for inclusion and exclusion criteria). Recruitment strategies involved contacting professional networks and organisations such as the Chartered Society of Physiotherapy, UK and the Australian Physiotherapy Association, along with the use of social media platforms (e.g., Facebook, X [formerly Twitter], and LinkedIn). Key accounts and influencers within MSK professional groups were tagged, and relevant hashtags (e.g., #msk, #fcp) were utilised. Additionally, a snowball sampling approach [31] was employed to engage physiotherapists working in first‐contact roles within the UK primary care and Australian emergency department. Due to the survey's exploratory nature and a lack of precise number of physiotherapists providing FCPS in the UK and Australia, the sample size was estimated based on the number of advanced practice physiotherapists in each country and prior national evaluation response rate. In the UK, approximately 10,000 physiotherapists work at the advanced practice level [32], with an estimated 5% (*n* = 500) in FCPS roles in primary care. A 30% response rate was anticipated, aiming for 150 participants. In Australia, 2,319 physiotherapists work at the advanced practice level, with an estimated 20% (*n* = 464) in FCPS roles in emergency departments [33]. A 30% response rate was also expected, aiming for 139 participants, resulting in an anticipated total sample of 289 across both countries. The survey provided a sampling frame for the Phase 2 study.

**Table 2:** Inclusion and exclusion criteria for phase 1 & 2 studies.

	Inclusion		Exclusion	
	UK	Australia	UK	Australia
Phase 1 Quantitative (Survey)	Physiotherapists majorly practicing in a first contact role		Physiotherapists majorly practicing in a secondary contact role	
	Majorly treat/manage patients with musculoskeletal conditions		Majorly treat/manage patients with other health conditions	
	In NHS primary care (General practice)	In Government hospital emergency department	Non‐NHS primary and/or community care	Nongovernment hospital emergency department
	Willingness to participate		Unwilling to participate	
Phase 2 Case study sites	NHS primary care providers with FCPS	Government hospital emergency departments with a FCPS	Non‐NHS primary and/or community care providers	Nongovernment hospital emergency department and government hospitals without a FCPS in Emergency department
	Willingness to engage in research		Unwilling to engage in research	
Managerial and research & innovation approval for case site participation.		No approval for site participation	
Case study sites participants ***(Physiotherapists)***	As in phase 1 (survey)		As in phase 1 (survey)	

Case study sites participants *****(Stakeholders [Team members])*****	GPs/Doctors, nurses, support staff, allied health and practice/strategic managers with knowledge/experience of FCPS		Team members that are not familiar with, and knowledgeable about FCPS	
	Non‐health care professionals such as IT staff and/or administrative staff with knowledge/experience of FCPS		Non‐health care professionals such as IT staff and/or administrative staff without the knowledge/experience of FCPS	
	Willingness to participate		Unwilling to participate	
Case study sites participants *****(Stakeholders [Patients])*****	Patients seen at their general practice by a physiotherapist due to musculoskeletal complaints	Patients seen at the emergency department by a physiotherapist due to musculoskeletal complaints	Patients seen at their general practice by other healthcare professionals and/or for other health complaints	Patients seen at the emergency department by other healthcare professionals and/or for other health complaints

	Capacity to make an informed decision		Lack capacity to make informed decision	
	Willingness to participate		Unwilling to participate	

#### 2.3.2 Phase 2: Qualitative (Case Studies)

Physiotherapists who express interest in participating in the follow‐up study and provide their email addresses during the survey were contacted. FCPS are based in different care settings, and variations in both adaptation process and timing are inevitable in the UK and Australia. Therefore, the sample was purposefully selected to include different service delivery adaptation approaches, considering the timing of adaptation. FCPS who experienced adaptation earlier in the pandemic were prioritised, as this enhanced learning on the rapidness element of the adaptation. Four case sites were purposefully selected across the UK and Australia, (UK *n* = 2, Australia *n* = 2). Recruiting four cases ensured the lead researcher (OA) could study each site in sufficient detail. While the heterogeneous sample enhanced a higher degree of saturation [34], data saturation may occur at ten to twelve interviews when considering an in‐depth interview [35], however more may be required for a heterogeneous sample such as this study [34]. Therefore, forty participants (10 from each case site) including physiotherapists providing FCPS for patients with MSK conditions at the case site, and other stakeholders (*individuals or groups involved in or affected by health and healthcare‐related decisions, programs, or policies, including the service providers and users*), were purposively selected based on their experience with FCPS during the COVID‐19 pandemic. Physiotherapists further assisted in participant recruitment by nominating other potential participants while OA formally recruited and obtained consent from them (See Table 2 for inclusion and exclusion criteria, including lists of FCPS stakeholders).

#### 2.3.3 Phase 3 (Stakeholders and Expert's Feedback)

A subset of physiotherapists in FCPS roles who previously participated in the study and expressed willingness to engage further, along with implementation and public health experts from institutions of study in the UK and Australia, will be contacted. Feedback will be gathered from those who agree to participate.

##### 2.3.3.1 Consent to Participate

Participation in this study is entirely voluntary. For Phase 1 (survey), the initial page introduced the study and provide detailed information through an embedded online participant information sheet and a consent form. Online consent was required to proceed with the survey. Participants could withdraw at any time and able to notify the research team within 1 month of completing the questionnaire if they wished to withdraw their data. All responses were anonymous except participants who voluntarily provided their contact details to receive a summary of the study or express interest in participating in future studies. Physiotherapists who provided their personal information were contacted by OA regarding Phases 2 of the study. OA formally obtained consent from these physiotherapists and other eligible participants (stakeholders) who were nominated. Potential participants received a participant information sheet and consent form via email, and given 2 weeks to decide on their participation [36]. Ongoing consent was sought. Data collected were non‐identifiable, with the same withdrawal opportunity for all participants as mentioned above. Phase 3 (consultation for feedback) will follow similar process.

##### 2.3.3.2 Ethical Considerations

For Phase 1, favourable ethical opinion was obtained from the Research governance office, at the University of Surrey UK (FHMS 21‐22 136 EGA) and Further approval was received from the Human Research Ethics committee at the University of Wollongong (UOW), Australia (UOW22/163). For Phase 2 and 3 of the study, ethics approval has been obtained from the Health Research Authority, UK for UK data (23/NW/0200), and Human Research Ethics Committee at the University of Wollongong (UOW), for Australia data (2023/ETH00538).

### 2.4 Data Collection

#### 2.4.1 Phase 1: Quantitative

A cross‐sectional online survey explored adaptations in FCPS during the COVID‐19 pandemic in the UK and Australia. Survey data was collected in the first half of 2023. The survey addressed research objectives 1 to 4 as shown in Table 1. Findings from this study provided a general understanding of the timing, type and process of changes that occurred in FCPS during the COVID‐19 pandemic including levels of FCPS readiness, responsiveness, and potential sustainability of these adaptations. It highlighted variations in each country's rapid adaptation processes. Responses from this study phase informed the phase 2 study focus.

#### 2.4.2 Survey Development

Due to the lack of a survey instrument for capturing rapid adaptation in healthcare services delivery, including FCPS during public health emergencies, a survey was developed by the lead researcher, OA, who is a PhD researcher and a physiotherapist, with the support of the supervisory team (TC, KS, NC, EP and VC) in 2023, using a combination of frameworks and findings from the literature [37]. It is comprised of 45 questions over three sections. Section one capturing changes in FCPS was guided by framework for reporting adaptation and modification enhancement (FRAME) framework [38], while section two addressing readiness and responsiveness was captured using findings from the literature and WHO responsiveness framework [39] respectively. Section three collects background information data and were asked about their willingness and interest to take part in the future study. The survey was transferred into Qualtrics. To help make the online survey more effective in measuring the responses on questions, varied question types have been used including ordinal, nominal, and ratio data including dichotomised questions, free‐text, single or multiple choice and Likert scales [40]. Two versions of the questionnaire were created with similar contents but cater for care contexts in the UK and Australia. It also enhances compliance with data sharing and management agreements.

#### 2.4.3 Survey Piloting

To test for content and face validity [37, 41], OA sent out the survey link to 12 physiotherapists providing FCPS in the UK and Australia for piloting, of which 8 responses were received. These individuals have been previously consulted during the early days of the study development. They were sent an email invitation along with the URL to the survey link. They were asked to complete an evaluation pro forma to address the appropriateness of the survey content, clarity, time taken to complete, completion ease and general comment. Completion time and unit completion rate was 13.9 (SD 10.0) minutes and 100%, respectively. Participants indicated that the questionnaire content addressed the research question and was relevant to FCPS. Suggestions to amend the terminology in six questions and to enhance the clarity about the time frame being referred to were made. To further clarify the issues around the time frame used in the questionnaire, two participants were consulted by telephone, and inconsistency with the timing was pointed out. Following team discussion, final modifications were made to the questionnaires (See Supporting Information file 1 for questionnaire).

#### 2.4.4 Phase 2: Qualitative

A multiple case study design was adopted to explain findings from the Phase 1 study in a deeper and more contextualised way [42, 43]. FCPS are spread over the UK and Australia and located in different healthcare settings which may also mean that the adaptations during COVID‐19 pandemic within these settings may differ. Therefore, multiple cases were selected across both countries based on varied types of adaptation reported in Phase 1 of the study to address research objectives 5 and 6. At each of the case study sites, one semi‐structured interview was conducted by OA with each physiotherapist and other stakeholders to provide in‐depth explanations about the findings from Phase 1 of the study. This commenced in the last quarter of 2023 and continued through May 2024. The interview guide was designed to provide insights into the opportunities created during the pandemic but also the challenges faced, and the strategies employed to overcome them. In addition, to describe and understand better the emerging patterns and type of changes documented from the survey data in Phase 1, and to further contextualise interview data, documents from healthcare organisations that discuss the aims, processes and outcomes of changes implemented in FCPS were also collected and analysed. A pilot sample was selected to inform development of the interview guide from the Phase 1 data.

#### 2.4.5 Phase 3: Stakeholders and Expert's Feedback

An email will be sent to physiotherapists in FCPS roles, as well as implementation and public health experts, requesting their feedback and recommendations on the rapid adaptation guide using a bespoke template developed apriori. This will address research objective 7, anticipated to commence in March 2025.

### 2.5 Data Analysis

#### 2.5.1 Phase 1: Quantitative

The statistical analysis assessed data completeness and identified quality issues. It includes descriptive statistics, summarising participant demographics, the timing of reported changes, and key changes implemented, providing a basis for comparison between the UK and Australia. Continuous data with a normal distribution will be presented as mean ± standard deviation, while non‐normally distributed data will be presented as median and interquartile range. Categorical variables will be presented as frequencies and percentages. For continuous variables with normally distributed data, a two‐way Analysis of Variance (ANOVA) assesses the main and interaction effects of timing and country on variables measuring changes in FCPS, readiness, and responsiveness. For non‐normally distributed variables (not a continuous scale), Generalised Linear Models (GLM) will be considered [44, 45]. Content analysis will be applied to analyse responses to open‐ended survey questions [45]. Both Microsoft Excel and IBM SPSS will be utilised to create data visualisations. Bar charts have been chosen to display frequencies of categorical variables, as they are widely recommended for their clarity and simplicity. Additionally, tables will be employed to present findings involving multiple variables in a structured format, enabling healthcare providers and policymakers to easily observe trends and changes in FCPS practices during the COVID‐19 pandemic [46, 47].

A two‐sided *p* < 0.05 will be considered statistically significant. All analyses are conducted using SPSS version 28© (IBM). Statistical and methodological reporting will follow SAMPL guidelines [48].

#### 2.5.2 Phase 2: Qualitative

Audio recordings transcribed verbatim. Computer‐Assisted Qualitative Data Analysis Software (CAQAS) NVivo Version 14 will be used to organise and manage the analysis. Interview data will be analysed by OA, with the support of the supervision team, in the context of healthcare documents that address the aims, processes, and outcomes of changes implemented in each FCPS case site. A case‐based, cross‐case analytical strategy that iteratively summarises cases around an inductive theme [49, 50] will be utilised. Thematic analysis will be used to draw out cross‐cutting themes, as it is flexible, not dependent on any theory or epistemology, permitting explanatory conclusions clustered around themes [51]. Reflexivity will be practised throughout the data collection and analysis process [45, 52].

#### 2.5.3 Phase 3: Mixed‐Methods Integration, Stakeholders and Experts' Feedback

The integration of mixed methods includes the synthesis of quantitative and qualitative findings and the development of recommendations for rapid adaptation. Pragmatism has been chosen as the paradigm for the analysis and interpretation of the findings from both quantitative and qualitative data [53, 54]. Although the mixed‐methods design uses a sequential approach with quantitative data guiding the qualitative study focus, pragmatism provides the opportunity to interpret findings from both perspectives. With the support of the supervision team, OA will iteratively analyse quantitative and qualitative data using abduction and data intersubjectivity, enhancing the transferability of findings [54, 55]. This will inform the development of preliminary context‐based rapid adaptation recommendations for FCPS, which can be applied in similar healthcare contexts, aligning with the WHO rapid adaptation operational guide. Feedback and recommendations sought from FCPS stakeholders, implementation and public health experts from the UK and Australia will enhance the development of a rapid adaptation recommendations.

### 2.6 Methodological Rigour

#### 2.6.1 Rigour, Validity, and Trustworthiness

Rigour was ensured in the planning and design of this study by using appropriate research tools (i.e., methodology, methods and frameworks) to meet the stated research objectives. In a mixed‐methods study, validity refers to the quality of inferences the researcher makes using both inductive and deductive reasoning [56]. This will be achieved in this study through questionnaire design, recruitment strategy, piloting, and research team meetings. The trustworthiness of the research will be determined by the researcher's activities, including confirmation of data interpretation with stakeholders, experts, the research team (i.e. intercoder reliability) and reflexivity. Reflexivity will be practiced throughout the study as it helps in appraising oneself, eliminate bias during research activities [51, 52].

### 2.7 Data Management

Data is being managed and processed in each country and stored on password‐protected computers, accessible only by team members and managed in accordance with current data protection regulations. Participants' contact details, provided to receive a summary of study findings or to participate in future studies, are stored in a separate password‐protected file in each country. All data stored on each country's institutional server are accessible to team members from that country, while OA, the lead researcher affiliated with institutions from both countries, has access to data from both countries. When needed, team members from other countries may have temporary access to non‐identifiable data. All data will be destroyed at the end of the 10‐year archive period. Research data will be kept with the strictest confidence, in compliance with both countries' Data Protection Regulations.

### 2.8 Dissemination of Findings

Study findings will be disseminated through physiotherapy events and professional organisations, including the Chartered Society of Physiotherapy UK and the Australian Physiotherapy Association, as well as international platforms like World Physiotherapy and other health services platforms. The results will also be shared via publication in academic peer‐reviewed journals and conference presentations. A lay summary will be provided to participants who request this information.

## 3 Discussion

The overall purpose of this study is to explore the rapid adaptation of FCPS for patients with MSK conditions during the COVID‐19 pandemic in the UK and Australia. Given the lack of evidence in this area, a sequential program of work is planned through the lenses of readiness, responsiveness, and sustainability, leading to informed recommendations for future rapid adaptation planning. The mixed‐methods research will provide a rigorous and rich contextualised account of FCPS rapid adaptation, providing key learnings that can be applied to implement evidence‐informed rapid adaptation in primary care and emergency departments FCPS during public health emergencies. This line of research will add useful perspective by providing an insight into the term referred to in implementation science as'rapid implementation' during public health emergencies and understand the impact of the rapid adaptation on responsiveness to patient's healthcare needs. It also aims to provide understanding about the barriers and facilitators for essential services implementing rapid adaptation, strategies utilised during the pandemic, with the potential to develop a recommendation that could prepare and guide FCPS and other similar healthcare services implement rapid adaptation successfully in case of future occurrence, mitigating potential disruptions and risks.

As the proposed study targets two countries with different contexts and health systems, it is important to account for these variations when interpreting the findings. Additionally, this study explores retrospective experiences, which may be susceptible to recall bias. However, given the profound impact of the pandemic on individuals, this bias might be minimised [57]. Moreover, the survey used event timelines to prompt respondents' memories, helping them contextualise their perspectives and experiences within a specific timeframe. Preliminary piloting of the survey demonstrated that participants could adequately recall and articulate their experiences. To further minimise recall bias and assess the consistency between self‐reported data and other sources, we will employ complementary strategies. These include analysing and triangulating participant responses with documents from healthcare organisation that detailed the aims, processes, and outcomes of the implemented changes within the FCPS. Lack of funding for incentives may have limited the number or quality of supporting documents obtained, considering participants' burden of accessing documents from their organisation. Since participation was entirely voluntary, some respondents might have been unwilling or unable to search for and provide relevant documents, potentially leading to less comprehensive data. However, findings will be further triangulated with evidence from existing literature and validated with stakeholders and experts [58].

## Author Contributions

**Oluwatoyin Adenike Adeniji:** conceptualization, methodology, investigation, funding acquisition, writing – original draft, writing – review and editing, resources, visualization, project administration, formal analysis, data curation. **Nicola Carey:** conceptualization, supervision, writing – review and editing, validation. **Evangelos Pappas:** validation, writing – review and editing, supervision. **Victoria Traynor:** supervision, validation, writing – review and editing. **Karen Stenner:** writing – review and editing, validation, supervision. **Theopisti Chrysanthaki:** conceptualization, validation, writing – review and editing, supervision.

## Ethics Statement

For Phase 1, favourable ethical opinion has been obtained from the Research governance office, at the University of Surrey UK (FHMS 21‐22 136 EGA) and Further approval was received from the Human Research Ethics Committee at the University of Wollongong (UOW), Australia (UOW22/163). For Phases 2 and 3 of the study, ethics approval has been obtained from the Health Research Authority, UK for UK data (23/NW/0200), and Human Research Ethics Committee through the University of Wollongong (UOW), for Australia data (2023/ETH00538).

## Conflicts of Interest

The authors declare no conflicts of interest.

## Transparency statement

Oluwatoyin Adenike Adeniji, affirms that this manuscript is an honest, accurate, and transparent account of the study being reported, no important aspects of the study have been omitted. All authors have read and approved the final version of the manuscript, Oluwatoyin had full access to all of the data in this study and takes complete responsibility for the integrity of the data and the accuracy of the data analysis.

## Supporting information

Supporting file 1.

## Data Availability

The authors have nothing to report.
