# Supplementary material for: Exploration of Rapid Adaptation of First Contact Physiotherapy Services During the COVID‐19 Pandemic: A Three‐Phase Sequential Mixed‐Methods Study Protocol
Source: Health Sci Rep. 2025 Apr 18;8(4):e70653. doi: 10.1002/hsr2.70653 (PMC12006840; doi:10.1002/hsr2.70653)
Supplement: Supplementary file 1 — Supporting file 1. [file HSR2-8-e70653-s001.pdf]

UK

Start of Block: Introduction

Q1
Exploring changes to first contact physiotherapy services for musculoskeletal patients during COVID 19 Pandemic

This survey is for physiotherapists who have practiced in the UK as a First Contact Physiotherapist (FCP) in primary care for musculoskeletal (MSK) patients since March 2020.

A FCP is a physiotherapist who is based in or works for GP surgeries with an expertise in the assessment and management of MSK conditions.

You are eligible to complete this survey as a FCP regardless of your employment status; full-time or part-time. You are also eligible to complete this survey if you have worked in multiple practices or changed where you practiced as FCP since March 2020.

This survey should take approximately 15 minutes to complete. This survey consists of three sections, most questions require a tick box response to indicate your answer. You can scroll backwards and forwards at any stage of the survey if you wish to review/change a previous response. You will be notified once you have reached the end of the survey. If you do not progress to the end of the survey and/or submit your responses, no data will be collected.

If you have any queries about this research, please contact me:

Participant information sheet

Before commencing this survey, please read the participant information sheet that can be accessed via the link below for further information about this research.

End of Block: Introduction

Start of Block: Consent

Q2 *After reading the participant information sheet, if you consent to participate in this survey, please acknowledge your agreement to the following statements by selecting 'yes' below if you wish to participate. If you do not agree, please select 'no' and submit, this will lead you to the end of the survey.*

I confirm that I have read and understood the participant information sheet attached above.

I understand that this survey is anonymous, and my participation is voluntary.

I understand that my personal data will only be collected if I indicate interest in participating in future research and that I am free to withdraw at any point, up to one month after completing the questionnaire.

I understand that the withdrawal of data could be done by contacting the researcher through the email address provided and I also understand that I need to provide my ID code to withdraw my data.

I understand that information I provide may be subject to review by responsible individuals from the University and/or regulators for monitoring and audit purposes.

I understand that information I provide will be used in various anonymised outputs, including report, publication, presentation, website and social media.

I understand that my personal data, including this consent form, which link me to the research data, will be kept securely in accordance with data protection guidelines, and only be accessible to the immediate research team or responsible persons at the University.

I understand any personal contact details collected about me, such as my name and email address, will not be shared beyond the research team.

I agree to take part in this study.

○ Yes (1)

○ No (2)

Skip To: End of Survey If After reading the participant information sheet, if you consent to participate in this survey, pl... = No

End of Block: Consent

Start of Block: Section 1

Q3 **Section 1**

Changes to FCP healthcare services delivery during the COVID 19 pandemic
COVID 19 has affected healthcare services since its declaration as a global pandemic in March 2020, leading to changes in service delivery. This section aims to explore what/ if any changes have occurred since March 2020 in the FCP service you work/worked for.

Changes occurred at different times for healthcare services. For some, changes occurred in the first three months of the COVID 19 pandemic while it took more months for others. The responses you provide should be based on the first changes that occurred in your FCP service delivery for MSK patients during the pandemic.

Q4 Was your FCP service suspended at any point due to the COVID 19 pandemic?

- ◯ Yes (14)
- ◯ No (15)
- ◯ I do not know (17)

Skip To: Q6 If Was your FCP service suspended at any point due to the COVID 19 pandemic? = No

Skip To: Q6 If Was your FCP service suspended at any point due to the COVID 19 pandemic? = I do not know

---

Q5 If yes, when was it suspended?

- ◯ Within the first 3 months into the pandemic (March 2020 to June 2020) (25)
- ◯ Between 4 to 6 months into the pandemic (July 2020 to September 2020) (20)
- ◯ After 6 months into the pandemic (October 2020 to March 2021) (21)
- ◯ After 12 months into the pandemic (April 2021 to October 2021) (22)
- ◯ After 18 months into the pandemic (November 2021 till present) (23)

Page Break

Q6 Are you aware of any changes to how your FCP service was delivered to MSK patients during the COVID 19 pandemic?

- ○ Yes (25)
- ○ No (26)
- ○ I am not sure (27)

Skip To: End of Survey If Are you aware of any changes to how your FCP service was delivered to MSK patients during the COV... = No

Page Break

Q7 Please indicate when the first changes occurred in your FCP service delivery during COVID 19 pandemic?

- ○ within the first 3 months into the pandemic (March 2020 to June 2020) (25)
- ○ between 4 to 6 months into the pandemic (July 2020 to September 2020) (20)
- ○ after 6 months into the pandemic (October 2020 to March 2021) (21)
- ○ after 12 months into the pandemic (April 2021 to October 2021) (22)
- ○ after 18 months into the pandemic (November 2021 till present) (23)

Page Break

Q8 The following set of questions will explore changes to access, mode of delivery, condition managed, interventions provided, patient case load, and administrative duties in your FCP service. The responses you provide should be based on the first changes that occurred in your FCP service delivery for MSK patients during the pandemic.

Page Break

Q9 ***Access***

Q10 Please indicate the use of the following ${Q7/ChoiceGroup/SelectedChoices}

	Increased (6)	Decreased (7)	Same (8)	Never used (9)	I cannot remember (10)
Triage system (26)	☐	☐	☐	☐	☐
In person self-booking (30)	☐	☐	☐	☐	☐
Online self-booking system (40)	☐	☐	☐	☐	☐
Self-referral (41)	☐	☐	☐	☐	☐
Ambulance service (42)	☐	☐	☐	☐	☐
FCP self-select from patient's list (39)	☐	☐	☐	☐	☐
Walk in (43)	☐	☐	☐	☐	☐
Other (If access option is not included in the list, please specify) (38)	☐	☐	☐	☐	☐

Q13 ***Mode of delivery***

Q14 Please indicate the use of the following ${Q7/ChoiceGroup/SelectedChoices}

	Increased (6)	Decreased (7)	Same (8)	Never used (9)	I cannot remember (10)
Face to face / in person (26)	☐	☐	☐	☐	☐
Remote (using telehealth) (27)	☐	☐	☐	☐	☐
Hybrid -- face to face/in-person and remote (using telehealth) (28)	☐	☐	☐	☐	☐

Q17 ***MSK Conditions managed***

Q18 Please indicate the extent to which these MSK conditions were managed.
${Q7/ChoiceGroup/SelectedChoices}

	Increased (6)	Decreased (7)	Same (8)	Never managed (9)	I cannot remember (10)
Soft tissue injuries e.g. sprain & strain (26)	☐	☐	☐	☐	☐
Arthritis (39)	☐	☐	☐	☐	☐
Spine-related pain (40)	☐	☐	☐	☐	☐
Dislocation/ minor fractures (41)	☐	☐	☐	☐	☐
Post orthopaedic surgical conditions (42)	☐	☐	☐	☐	☐
Muscle or ligament pain (43)	☐	☐	☐	☐	☐
Other (please specify) (38)	☐	☐	☐	☐	☐

Q21 ***Interventions provided***

Q22 Please indicate how the use of the following changed ${Q7/ChoiceGroup/SelectedChoices}

	Increased (6)	Decreased (7)	Same (8)	Never used (9)	Not applicable (11)	I cannot remember (12)
Self-management advice (26)	☐	☐	☐	☐	☐	☐
Referral to other medical specialists (30)	☐	☐	☐	☐	☐	☐
Referral to physiotherapy services (31)	☐	☐	☐	☐	☐	☐
Referral to the community services (32)	☐	☐	☐	☐	☐	☐
Application of orthotic devices or cast (33)	☐	☐	☐	☐	☐	☐
Booking for surgery (34)	☐	☐	☐	☐	☐	☐
Prescribing Imaging (35)	☐	☐	☐	☐	☐	☐
Prescribing medication (36)	☐	☐	☐	☐	☐	☐
Giving Injection (37)	☐	☐	☐	☐	☐	☐
Other (please specify) (38)	☐	☐	☐	☐	☐	☐

Q25 ***Patient caseload***

Q26 Please indicate how your patient caseload changed ${Q7/ChoiceGroup/SelectedChoices}

	Click to write Column 1	Click to write Column 2	Click to write Column 3	Click to write Column 4	Click to write Column 5	Click to write Column 6
	Increased (1)	Decreased (1)	Same (1)	Please indicate the average number or time (in minutes) (1)	Not applicable (1)	I cannot remember (1)
Average number of MSK patients per week (26)	☐	☐	☐		☐	☐
Average number of appointments per MSK patient (30)	☐	☐	☐		☐	☐
Average consultation time for MSK patients (32)	☐	☐	☐		☐	☐

Page Break

Q33 ***Administrative and/or managerial duties***

Q34 Please indicate how your administrative/managerial duties changed ${Q7/ChoiceGroup/SelectedChoices}

- ◯ Increased (40)
- ◯ Decreased (41)
- ◯ Same (43)
- ◯ I cannot remember (44)

End of Block: Section 1

Start of Block: Section 2

Q38 **Section 2**

Readiness and responsiveness of FCP healthcare service delivery for MSK patients during COVID 19 pandemic.
This section seek to understand your perception of the ability of your organisation to manage the challenges and needs that the pandemic presented.

Page Break

Q39 I believe my FCP service was ready for the first changes that occurred in terms of:

	Strongly disagree (1)	Disagree (2)	Neither agree nor disagree (3)	Agree (4)	Strongly agree (5)	I do not know (6)	Not applicable to FCP setting (14)
Financial resources available i.e., emergency funding (1)	▢	▢	▢	▢	▢	▢	▢
Human resources i.e., enough staff (5)	▢	▢	▢	▢	▢	▢	▢
Human resources management i.e., staff training (6)	▢	▢	▢	▢	▢	▢	▢
Infrastructure i.e., space, technology equipment (7)	▢	▢	▢	▢	▢	▢	▢
Technical support (14)	▢	▢	▢	▢	▢	▢	▢
Leadership support and motivation for change (8)	▢	▢	▢	▢	▢	▢	▢
Mission and vision i.e., have clear norms and values of how things are done (15)	▢	▢	▢	▢	▢	▢	▢
Internal communication, i.e., access to up-to-date information within practice/hospital (9)	▢	▢	▢	▢	▢	▢	▢

External partnerships and communication i.e., government agencies, patients, NGOs (4)	☐	☐	☐	☐	☐	☐	☐
Strategy for organisational learning is in place i.e., measure and evaluation (13)	☐	☐	☐	☐	☐	☐	☐
Supplies and procurement i.e., PPE, (16)	☐	☐	☐	☐	☐	☐	☐
Availability of crises response guidelines, regulations, disaster response plan (12)	☐	☐	☐	☐	☐	☐	☐
Compatibility of telehealth with practice (10)	☐	☐	☐	☐	☐	☐	☐
Outputs, service, and results i.e., Increased demand for musculoskeletal services during Covid 19 pandemic (11)	☐	☐	☐	☐	☐	☐	☐
Stakeholder's buy-in (17)	☐	☐	☐	☐	☐	☐	☐

Q40 I believe my FCP services met patient's expectations and needs when first changes occurred in terms of:

	Strongly disagree (2)	Disagree (3)	Neither agree nor disagree (4)	Agree (5)	Strongly agree (6)	I do not know (7)	Not applicable to FCP setting (8)
**Prompt attention** In terms of timeliness of care and due attention (1)							
**Prompt attention** In terms of having short waiting times for investigations like Xray, MRI, blood test (28)							
**Choice** Patients being able to voluntarily choose FCP services for their MSK conditions (19)							
**Choice** Patient being able to choose between in-person care and virtual care (30)							
**Amenities** Convenience of facilities (20)							
**Autonomy** (Patient's involvement in medical decision making) (21)							
**Communication** Patient's interactions with service providers (Listening to patients carefully, explaining things to patient and allow time to ask questions) (23)							

**Respect** Dignity and treatment with regard (24)	▢	▢	▢	▢	▢	▢	▢
**Confidentiality** keeping medical secrets and maintaining privacy (25)	▢	▢	▢	▢	▢	▢	▢
**Access to social support** (Family, friends) (2)	▢	▢	▢	▢	▢	▢	▢
**Equitability** (Equal access to care across all races, gender, socioeconomic status, georgrpahical locations (i.e. suburban vs rural), MSK conditions). (22)	▢	▢	▢	▢	▢	▢	▢

End of Block: Section 2

Start of Block: Section 3

Q41 **Section 3**

Background information

This section will collect basic demographic and background information on your FCP practice since the pandemic i.e., since March 2020

Q42
In which UK country have you primarily practiced as an FCP since the start of COVID 19 pandemic?

- England (1)
- Scotland (2)
- Wales (3)
- Northern Ireland (4)

Display This Question:

If In which UK country have you primarily practiced as an FCP since the start of COVID 19 pandemic? = England

Q43 What geographical region do you primarily work? If multiple, select the geographical region that you have spent the most of your time since March 2020

- East of England (1)
- London (12)
- Midlands (13)
- North East and Yorkshire (14)
- North West (15)
- South East (16)
- South West (17)

Display This Question:

If In which UK country have you primarily practiced as an FCP since the start of COVID 19 pandemic? = Scotland

Q44 What geographical region do you primarily work? If multiple, select the geographical region that you have spent the most of your time since March 2020

- ◯ Eastern Scotland (1)
- ◯ Highlands and Islands (4)
- ◯ North Eastern Scotland (5)
- ◯ South Western Scotland (6)
- ◯ Other (please specify) (7) ____

Display This Question:

If In which UK country have you primarily practiced as an FCP since the start of COVID 19 pandemic? = Wales

Q45 What geographical region do you primarily work? If multiple, select the geographical region that you have spent the most of your time since March 2020

- ◯ East Wales (1)
- ◯ West Wales (6)
- ◯ Other (please specify) (7) ____

Display This Question:

If In which UK country have you primarily practiced as an FCP since the start of COVID 19 pandemic? = Northern Ireland

Q46 What geographical region do you primarily work? If multiple, select the geographical region that you have spent the most of your time since March 2020

- Belfast (1)
- East of Northern Ireland (4)
- North of Northern Ireland (5)
- West and South of Northern Ireland (6)
- Outer Belfast (7)
- Other (please specify) (8) ____

Q47 When did FCP services start in your practice?

- ≥10 years prior to Covid 19 pandemic (1)
- 4 -9 years prior to Covid 19 pandemic (4)
- ≤3 years prior to Covid 19 pandemic (5)
- During Covid 19 pandemic (6)
- I do not know (7)

Q48 How many years of experience do you have as a physiotherapist before COVID 19 pandemic?

- ≥10 years (1)
- 5 - 9 years (4)
- 2 - 4years (5)
- < 2 years (6)
- I do not have prior experience (8)

Q49 How long have you worked as an FCP in primary care?

- ≥10 years (1)
- 5 - 9 years (9)
- 3 - 4 years (10)
- < 3 years (11)

Q50 What percentage of your clinical time is spent each week as an FCP treating MSK patients?

- 0-25% (153)
- 26-50% (154)
- 51-75% (155)
- 76-100% (156)

Q51 What is your highest academic qualification?

- ○ Diploma (1)
- ○ Bachelor’s degree (4)
- ○ Masters (5)
- ○ Doctorate (6)
- ○ Other (please specify) (7) ____

Q52 What is your gender

- ○ Female (1)
- ○ Male (4)
- ○ Non-binary/ third gender (5)
- ○ Would rather not say (3)
- ○ Other (please specify) (6) ____

Q53 What is your age group?

- ○ 18 to 24 (1)
- ○ 25 to 34 (4)
- ○ 35 to 44 (5)
- ○ 45 to 54 (6)
- ○ 55 to 64 (7)
- ○ 65 or over (8)

End of Block: Section 3

Start of Block: Thank you

Q54 **Thank you for completing this Survey**
You will have the opportunity to request for the summary of the study findings through the researcher's email address provided at the start of the survey, please get in touch if interested.

Q55 Please indicate if you would be interested in being contacted for future research.

- ◯ Yes, I agree to be contacted by the researcher regarding participation in future research (4)
- ◯ I do not wish to take part in future research (7)

Skip To: Q59 If Please indicate if you would be interested in being contacted for future research. = I do not wish to take part in future research

---

Display This Question:

If Please indicate if you would be interested in being contacted for future research. = Yes, I agree to be contacted by the researcher regarding participation in future research

Q56 Please use the boxes below to supply your contact details:

(Your personal data will be held and processed in the strictest confidence, and in accordance with current data protection regulations. It will not be published in the survey results).

Name
Q57 Email address
Q58 Work Postcode
Q59 **This is the end of the survey**

If you would like to make any comments or suggestions about the survey you have just completed, please feel free to do so in the box below.

Please click on the next button to submit your responses

AUSTRALIA

Start of Block: Introduction

Q1

Exploring changes to first contact physiotherapy services for musculoskeletal patients during COVID 19 Pandemic

This survey is for physiotherapists who have practiced in Australian public emergency departments as first point of contact physiotherapist for musculoskeletal (MSK) patients since March 2020.

First point of contact physiotherapist (FCP) is a physiotherapist who is based in a public emergency department with an expertise in the assessment and management of MSK conditions.

You are eligible to complete this survey as FCP regardless of your employment status; full-time or part-time. You are also eligible to complete this survey if you have worked in multiple hospitals or changed the hospital where you practiced since March 2020.

This survey should take approximately 15 minutes to complete. This survey consists of three sections, most questions require a tick box response to indicate your answer. You can scroll backwards and forwards at any stage of the survey if you wish to review/change a previous response. You will be notified once you have reached the end of the survey. If you do not progress to the end of the survey and/or submit your responses, no data will be collected.

If you have any queries about this research, please contact me:

Participant information

Before commencing this survey, please read the participant information sheet that can be accessed via the link below for further information about this research.

End of Block: Introduction

Start of Block: Consent

Q2 *After reading the participant information sheet, if you consent to participate in this survey, please acknowledge your agreement to the following statements by selecting 'yes' below if you wish to*

participate. If you do not agree, please select 'no' and submit, this will lead you to the end of the survey.

I confirm that I have read and understood the participant information sheet attached above.

I understand that this survey is anonymous, and my participation is voluntary.

I understand that my personal data will only be collected if I indicate interest in participating in future research and that I am free to withdraw at any point, up to one month after completing the questionnaire.

I understand that the withdrawal of data could be done by contacting the researcher through the email address provided and I also understand that I need to provide my ID code to withdraw my data.

I understand that information I provide may be subject to review by responsible individuals from the University and/or regulators for monitoring and audit purposes.

I understand that information I provide will be used in various anonymised outputs, including report, publication, presentation, website and social media.

I understand that my personal data, including this consent form, which link me to the research data, will be kept securely in accordance with data protection guidelines, and only be accessible to the immediate research team or responsible persons at the University.

I understand any personal contact details collected about me, such as my name and email address, will not be shared beyond the research team.

I agree to take part in this study.

○ Yes (1)

○ No (2)

Skip To: End of Survey If After reading the participant information sheet, if you consent to participate in this survey, pl... = No

End of Block: Consent

Start of Block: Section 1

Q3 **Section 1**

Changes to APP healthcare services delivery during the COVID 19 pandemic
COVID 19 has affected healthcare services since its declaration as a global pandemic in March 2020, leading to changes in service delivery. This section aims to explore what/ if any changes have occurred since March 2020 in the FCP services you work/worked for.

Changes occurred at different times for healthcare services. For some, changes occurred in the first three months of the COVID 19 pandemic while it took more months for others. The responses you provide should be based on the first changes that occurred in your FCP service delivery for MSK patients during the pandemic.

Page Break

Q4 Was your FCP services suspended at any point due to the COVID 19 pandemic?

- ○ Yes (14)
- ○ No (15)
- ○ I do not know (17)

Skip To: Q6 If Was your FCP service suspended at any point due to the COVID 19 pandemic? = No

Skip To: Q6 If Was your FCP service suspended at any point due to the COVID 19 pandemic? = I do not know

Q5 If yes, when was it suspended?

- ○ Within the first 3 months into the pandemic (March 2020 to June 2020) (25)
- ○ Between 4 to 6 months into the pandemic (July 2020 to September 2020) (20)
- ○ After 6 months into the pandemic (October 2020 to March 2021) (21)
- ○ After 12 months into the pandemic (April 2021 to October 2021) (22)
- ○ After 18 months into the pandemic (November 2021 till present) (23)

Page Break

Q6 Are you aware of any changes to how your FCP service was delivered to MSK patients during the COVID 19 pandemic?

- ◯ Yes (25)
- ◯ No (20)
- ◯ I am not sure (26)

Skip To: End of Survey If Are you aware of any changes to how your FCP service was delivered to MSK patients during the COV... = No

Page Break

Page Break

Q7 Please indicate when the first changes occurred in your FCP service delivery during COVID 19 pandemic?

- ◯ within the first 3 months into the pandemic (March 2020 to June 2020) (25)
- ◯ between 4 to 6 months into the pandemic (July 2020 to September 2020) (20)
- ◯ after 6 months into the pandemic (October 2020 to March 2021) (21)
- ◯ after 12 months into the pandemic (April 2021 to October 2021) (22)
- ◯ after 18 months into the pandemic (November 2021 till present) (23)

Page Break

Q8 The following set of questions will explore changes to access, mode of delivery, condition managed, interventions provided, patient case load, and administrative duties in your FCP service. The responses you provide should be based on the first changes that occurred in your FCP service delivery for MSK patients during the pandemic.

Page Break

Q9 ***Access***

Q10 Please indicate the use of the following ${Q7/ChoiceGroup/SelectedChoices}

	Increased (6)	Decreased (7)	Same (8)	Never used (9)	I cannot remember (10)
Triage system (26)	▢	▢	▢	▢	▢
Online self-booking system (40)	▢	▢	▢	▢	▢
Ambulance service (42)	▢	▢	▢	▢	▢
FCP self-select from patient's list (39)	▢	▢	▢	▢	▢
Walk in (43)	▢	▢	▢	▢	▢
Other (If access option is not included in the list, please specify) (38)	▢	▢	▢	▢	▢

Q13 ***Mode of delivery***

Q14 Please indicate the use of the following ${Q7/ChoiceGroup/SelectedChoices}

	Increased (6)	Decreased (7)	Same (8)	Never used (9)	I cannot remember (10)
Face to face / in person (26)	☐	☐	☐	☐	☐
Remote (using telehealth) (27)	☐	☐	☐	☐	☐
Hybrid -- face to face/in-person and remote (using telehealth) (28)	☐	☐	☐	☐	☐

Page Break

Q17 ***MSK Conditions managed***

Q18 Please indicate the extent to which these MSK conditions were managed.
${Q7/ChoiceGroup/SelectedChoices}

	Increased (6)	Decreased (7)	Same (8)	Never managed (9)	I cannot remember (10)
Soft tissue injuries e.g. sprain & strain (26)	☐	☐	☐	☐	☐
Arthritis (39)	☐	☐	☐	☐	☐
Spine-related pain (40)	☐	☐	☐	☐	☐
Dislocation/ minor fractures (41)	☐	☐	☐	☐	☐
Post orthopaedic surgical conditions (42)	☐	☐	☐	☐	☐
Muscle or ligament pain (43)	☐	☐	☐	☐	☐
Other (please specify) (38)	☐	☐	☐	☐	☐

Q21 ***Interventions provided***

Q22 Please indicate how the use of the following changed ${Q7/ChoiceGroup/SelectedChoices}

	Increased (6)	Decreased (7)	Same (8)	Never used (9)	Not applicable (11)	I cannot remember (12)
Self-management advice (26)	☐	☐	☐	☐	☐	☐
Referral to other specialists (30)	☐	☐	☐	☐	☐	☐
Referral to physiotherapy services (31)	☐	☐	☐	☐	☐	☐
Referral to the community services (32)	☐	☐	☐	☐	☐	☐
Application of orthotic devices or cast (33)	☐	☐	☐	☐	☐	☐
Imaging (39)	☐	☐	☐	☐	☐	☐
Booking for Surgery (40)	☐	☐	☐	☐	☐	☐
Other (please specify) (38)	☐	☐	☐	☐	☐	☐

Q25 ***Patient caseload***

Q26 Please indicate how your patient caseload changed ${Q7/ChoiceGroup/SelectedChoices}

	Click to write Column 1	Click to write Column 2	Click to write Column 3	Click to write Column 4	Click to write Column 5	Click to write Column 6
	Increased (1)	Decreased (1)	Same (1)	Please indicate the average number or time (in minutes) (1)	Not applicable (1)	I cannot remember (1)
Average number of MSK patients per week (26)	☐	☐	☐		☐	☐
Average number of appointments per MSK patient (30)	☐	☐	☐		☐	☐
Average consultation time for MSK patients (32)	☐	☐	☐		☐	☐

Q33 ***Administrative and/or managerial duties***

Q34 Please indicate how your administrative/managerial duties changed ${Q7/ChoiceGroup/SelectedChoices}

- ◯ Increased (40)
- ◯ Decreased (41)
- ◯ Same (43)
- ◯ I cannot remember (44)

End of Block: Section 1

Start of Block: Section 2

Q38 **Section 2**

Readiness and responsiveness of FCP healthcare service delivery for MSK patients during COVID 19 pandemic.
This section seeks to understand your perception of the ability of your organisation to manage the challenges and needs that the pandemic presented.

Similar to Section 1, the responses you provide should be based on the first changes that occurred in your FCP service delivery for MSK patients during the pandemic.

Page Break

Q39 I believe my FCP service was ready for the first changes that occurred in terms of:

	Strongly disagree (1)	Disagree (2)	Neither agree nor disagree (3)	Agree (4)	Strongly agree (5)	I do not know (6)	Not applicable to FCP setting (14)
Financial resources available i.e., emergency funding (1)	☐	☐	☐	☐	☐	☐	☐
Human resources i.e., enough staff (5)	☐	☐	☐	☐	☐	☐	☐
Human resources management i.e., staff training (6)	☐	☐	☐	☐	☐	☐	☐
Infrastructure i.e., space, technology equipment (7)	☐	☐	☐	☐	☐	☐	☐
Technical support (14)	☐	☐	☐	☐	☐	☐	☐
Leadership support and motivation for change (8)	☐	☐	☐	☐	☐	☐	☐
Mission and vision i.e., have clear norms and values of how things are done (15)	☐	☐	☐	☐	☐	☐	☐
Internal communication, i.e., access to up-to-date information within practice/hospital (9)	☐	☐	☐	☐	☐	☐	☐

External partnerships and communication i.e., government agencies, patients, NGOs (4)	☐	☐	☐	☐	☐	☐	☐
Strategy for organisational learning is in place i.e., measure and evaluation (13)	☐	☐	☐	☐	☐	☐	☐
Supplies and procurement i.e., PPE, (16)	☐	☐	☐	☐	☐	☐	☐
Availability of crises response guidelines, regulations, disaster response plan (12)	☐	☐	☐	☐	☐	☐	☐
Compatibility of telehealth with practice (10)	☐	☐	☐	☐	☐	☐	☐
Outputs, service, and results i.e., Increased demand for musculoskeletal services during Covid 19 pandemic (11)	☐	☐	☐	☐	☐	☐	☐
Stakeholder's buy-in (17)	☐	☐	☐	☐	☐	☐	☐

Q40 I believe my FCP service met patient's expectations and needs when first changes occurred in terms of:	Strongly disagree (2)	Disagree (3)	Neither agree nor disagree (4)	Agree (5)	Strongly agree (6)	I do not know (7)	Not applicable to FCP setting (8)
**Prompt attention** In terms of timeliness of care and due attention (1)	☐	☐	☐	☐	☐	☐	☐
**Prompt attention** In terms of having short waiting times for investigations like Xray, MRI, blood test (28)	☐	☐	☐	☐	☐	☐	☐
**Choice** Patients being able to voluntarily choose FCP services for their MSK conditions (19)	☐	☐	☐	☐	☐	☐	☐
**Choice** Patient being able to choose between in-person care and virtual care (30)	☐	☐	☐	☐	☐	☐	☐
**Amenities** Convenience of facilities (20)	☐	☐	☐	☐	☐	☐	☐
**Autonomy** (Patient's involvement in medical decision making) (21)	☐	☐	☐	☐	☐	☐	☐

**Communication** Patient's interactions with service providers (Listening to patients carefully, explaining things to patient and allow time to ask questions) (23)	☐	☐	☐	☐	☐	☐	☐
**Respect** Dignity and treatment with regard (24)	☐	☐	☐	☐	☐	☐	☐
**Confidentiality** keeping medical secrets and maintaining privacy (25)	☐	☐	☐	☐	☐	☐	☐
**Access to social support** (Family, friends) (2)	☐	☐	☐	☐	☐	☐	☐
**Equitability** (Equal access to care across all races, gender, socioeconomic status, georgrpahical locations (i.e. suburban vs rural), MSK conditions). (22)	☐	☐	☐	☐	☐	☐	☐

End of Block: Section 2

Start of Block: Section 3

Q41 **Section 3**

Background information

This section will collect basic demographic and background information on your FCP service since the pandemic i.e., since March 2020

Q42 In which state or territory have you primarily practice as a FCP since the start of the Covid 19 pandemic? If multiple, select the state or territory that you spent the most time working

- Australian Capital Territory (1)
- New South Wales (5)
- Northern Territory (6)
- Queensland (7)
- South Australia (8)
- Tasmania (9)
- Victoria (10)
- Western Australia (11)

Q43 Please provide the postocode for where you primarily worked. If multiple, please provide the postcode for where you spent the most time working

____

Q44 When did FCP services start in your hospital?

- ≥10 years prior to Covid 19 pandemic (1)
- 4 -9 years prior to Covid 19 pandemic (4)
- ≤3 years prior to Covid 19 pandemic (5)
- During Covid 19 pandemic (6)
- I do not know (7)

Q45 How many years of experience do you have as a physiotherapist before COVID 19 pandemic?

- ≥10 years (1)
- 5 - 9 years (4)
- 2 - 4years (5)
- < 2 years (6)
- I do not have prior experience (8)

Q46 How long have you worked as a FCP in emergency department?

- ≥10 years (1)
- 5 - 9 years (9)
- 3 - 4 years (10)
- < 3 years (11)

Q47 What percentage of your clinical time is spent each week as an FCP treating MSK patients?

- 0-25% (153)
- 26-50% (154)
- 51-75% (155)
- 76-100% (156)

Q48 What is your highest academic qualification?

- ○ Diploma (1)
- ○ Bachelor’s degree (4)
- ○ Masters (5)
- ○ Doctorate (6)
- ○ Other (please specify) (7) ____

Q49 What is your gender

- ○ Female (1)
- ○ Male (4)
- ○ Non-binary/ third gender (5)
- ○ Would rather not say (3)
- ○ Other (please specify) (6) ____

Q50 What is your age group?

- ○ 18 to 24 (1)
- ○ 25 to 34 (4)
- ○ 35 to 44 (5)
- ○ 45 to 54 (6)
- ○ 55 to 64 (7)
- ○ 65 or over (8)

End of Block: Section 3

Start of Block: Thank you

Q51 **Thank you for completing this Survey**

Please indicate if you are interested in receiving a summary of the survey findings and or if you would be interested in being contacted for future research.

- ○ Yes, I wish to receive a copy of the summary of the findings (4)
- ○ Yes, I agree to be contacted by the research team regarding participation in follow up studies (7)
- ○ Yes, to both of the above (8)
- ○ I do not wish to receive a summary nor take part in future research (9)

Skip To: Q54 If Thank you for completing this Survey Please indicate if you are interested in receiving a summar... = I do not wish to receive a summary nor take part in future research

Q52 Please use the boxes below to supply your contact details:

(Your personal data will be held and processed in the strictest confidence, and in accordance with current data protection regulations. It will not be published in the survey results).

Name
Q53 Email address
Q54 **This is the end of the survey**

If you would like to make any comments or suggestions about the survey you have just completed, please feel free to do so in the box below.

Please click on the next button to submit your responses
